# For Stutterers

**Published:** 1885-08

**Authors:** 


### For Stutterers.

A gentleman who stammered from childhood almost up to manhood gives a very simple remedy for the misfortune : "Go into a room where you will be quiet and alone, get some book that will interest but not excite you, and sit down and read two hours aloud to yourself, keeping your teeth together. Do the same thing every two or three days, or once a week if very tiresome, always taking care to read slowly and distinctly, moving the lips, but not the teeth. Then, when conversing with others, try to speak as slowly and distinctly as possible, and make up your mind that you will not stammer, Well, I tried this remedy, not having much faith in it, I must confess, but willing to do most anything to cure myself of such an annoying difficulty. I read for two hours aloud with my teeth together. The first result was to make my tongue and jaws ache, that is, while I was reading, and the next to make me feel as if something had loosened my talking apparatus, for I could speak with less difficulty immediately. The change was so great that everyone who knew me remarked it. I repeated the remedy every five or six days for a month, and then at longer intervals until cured."—*Phys. and Surg. Invest.*

